# Supplementary material for: Precision medicine for liver tumours with quantitative MRI and whole genome sequencing (Precision1 trial): study protocol for observational cohort study
Source: BMJ Open. 2022 Apr 4;12(4):e057163. doi: 10.1136/bmjopen-2021-057163 (PMC8984042; doi:10.1136/bmjopen-2021-057163)
Supplement: Author's manuscript [file bmjopen-2021-057163.draft_revisions.pdf]

BMJ Open

BMJ Open is committed to open peer review. As part of this commitment we make the peer review history of every article we publish publicly available.

When an article is published we post the peer reviewers' comments and the authors' responses online. We also post the versions of the paper that were used during peer review. These are the versions that the peer review comments apply to.

The versions of the paper that follow are the versions that were submitted during the peer review process. They are not the versions of record or the final published versions. They should not be cited or distributed as the published version of this manuscript.

BMJ Open is an open access journal and the full, final, typeset and author-corrected version of record of the manuscript is available on our site with no access controls, subscription charges or pay-per-view fees (http://bmjopen.bmj.com).

If you have any questions on BMJ Open's open peer review process please email info.bmjopen@bmj.com

BMJ Open

Precision medicine for liver tumours with quantitative magnetic resonance imaging and whole genome sequencing (Precision1 trial): Study protocol for observational cohort study.

Journal:	*BMJ Open*
Manuscript ID	bmjopen-2021-057163
Article Type:	Protocol
Date Submitted by the Author:	08-Sep-2021

Complete List of Authors:	Welsh, Fenella; Basingstoke and North Hampshire Hospital, Department of Hepatobiliary Surgery<br>Connell, John; Perspectum Diagnostics Ltd<br>Kelly, Matthew; Perspectum Diagnostics Ltd<br>Gooding, Sarah; University of Oxford<br>Banerjee, Rajarshi; Perspectum Diagnostics Ltd<br>Rees, Myrddin; Basingstoke and North Hampshire Hospital, Department of Hepatobiliary Surgery

Keywords:	Hepatobiliary surgery < SURGERY, Magnetic resonance imaging < RADIOLOGY & IMAGING, GENETICS

SCHOLARONE™
Manuscripts

Precision medicine for liver tumours with quantitative magnetic resonance imaging and whole genome sequencing (Precision1 trial): Study protocol for observational cohort study.

Fenella KS Welsh[1], John J Connell[2], Matt Kelly[2], Sarah Gooding[3], Rajarshi Banerjee[2], Myrddin Rees[1]

[1] Department of Hepatobiliary Surgery, Basingstoke and North Hampshire Hospital, Basingstoke, Hampshire, United Kingdom.

[2] Perspectum Ltd Gemini One, 5520 John Smith Drive, Oxford, United Kingdom.

[3] University of Oxford, Oxford, United Kingdom.

Corresponding author:

Dr John Connell

Perspectum Ltd

Gemini One, 5520 John Smith Drive

Oxford

For peer review only

OX4 2LL

john.connell@perspectum.com

+44 (0) 1865 655 343

For peer review only

Abstract

Introduction

Radiogenomic analysis of patients being considered for liver resection is seldom performed in the clinic despite recent evidence indicating that quantitative MRI could improve post-hepatectomy outcomes. Meanwhile, the increasingly accessible results from whole genome sequencing reporting on clinically actionable genetic biomarkers is yet to be fully integrated into the clinical care pathway.

Methods and analysis

A prospective observational cohort study of up to 200 participants is planned, recruiting adults with primary or secondary liver cancer being considered for liver resection at Hampshire Hospitals NHS Foundation Trust. The data will be evaluated to address the primary endpoint to calculate the proportion of participants in which the results from whole genome sequencing would have resulted in a change in clinical management. Participants will be offered an additional non-invasive quantitative MRI scan prior to the operation and the impact of the imaging results on treatment decision making will be evaluated.

Ethics and dissemination

This study was reviewed by the NHS Health Research Authority and given favourable opinion by the Brighton and Sussex Research Ethics Committee (REC Reference

For peer review only

For peer review only - http://bmjopen.bmj.com/site/about/guidelines.xhtml

20/PR/0222). Research findings will be discussed with a Patient and Public Involvement and Engagement group, presented at relevant scientific conferences and published in open access journals.

Trial Registration

Clinicaltrials.gov identifier: NCT04597710(1)

Strengths and limitations of this study

- Precision1 will be the first study examining the clinical utility of radiogenomic information during the surgical treatment planning stage for patients with primary and secondary liver cancer
- Our prospective study design will allow us to understand the extent to which quantitative MRI of the liver impacts surgical treatment planning along with the additional value of whole genome sequencing to help us to identify the presence of any clinically actionable genetic biomarkers which may support precision medicine provision to patients.
- The primary limitation of this study is the omission of a tightly matched negative control group where the impact of quantitative imaging on improving patient outcomes could be rigorously examined.

For peer review only

Introduction

The incidence of treatable liver tumours is on the rise globally, driven by obesity, viral hepatitis and metastases from colorectal cancers. Survival rates can be improved with optimised allocation of treatment options including surgical resection, radiofrequency ablation, embolisation, chemotherapy and targeted molecular therapies (including immunotherapy). The key motivation of this study is to help patients access the most suitable treatment combinations, by integrating radiogenomic(2) and clinical data.

A similar integrated approach, integrating radiology and pathology, has been shown to improve outcomes in breast cancer care(3). Detailed pathologic analysis of the surgical specimen from breast carcinoma biopsy provides valuable feedback to the radiologist, establishes the completeness of surgical intervention, and generates predictive information for therapeutic decisions(4).

Whole genome sequencing (WGS) has revealed cancer driver mutations and the complex molecular profile of liver cancer(5). In many metastatic solid tumours, WGS has been used to identify a significant patient population (31%) who present with a biomarker that predicts sensitivity to a drug and lack any known resistance biomarkers for the same drug(6). Identifying which patients possess genomic variants that predict drug response or resistance will allow clinicians to make the optimal treatment decisions. The next technological challenge is integrating WGS into scalable clinical practice.

For peer review only

In addition to the molecular profile of the patient's liver cancer, a non-invasive assessment of the overall health of the liver using quantitative MRI imaging will be carried out in this study. The MRI metrics from Liver*MultiScan*® can quantify changes to liver tissue characteristics, including fibro-inflammation and fat, with the accuracy and reproducibility of the results demonstrated in a number of studies(7–9). A recent InnovateUK grant supported Perspectum, Hampshire Hospitals NHS Foundation Trust (HHFT) and University of Edinburgh to assess the use of quantitative MRI in decision support for liver resection. This prospective 143 patient study showed quantitative MRI using Hepatica™ (which incorporates Liver*MultiScan*®) can predict length of patient stay(10–12). Patients with liver cancer plus often undiagnosed chronic liver disease performed worse than those with healthy native livers; they also recovered more slowly, costing an extra 1.5 days of hospital stay per patient, a significant clinical impact.

The gold standard of liver assessment relies on imaging and biopsies. Advances in quantitative algorithms provide richer data about the cellular changes associated with liver disease, and greater insight into disease monitoring and treatment efficacy from MRI and digital pathology. In this project, by additionally analysing the genome of both tumour and germline, we aim to understand which variants are driving tumour growth and potentially amenable to targeted therapies and impacting treatment response, along with variants associated with response to surgery and liver regeneration.

For peer review only

In previous work we have demonstrated the ability to combine MRI imaging, liver histopathology, and circulating (enzyme) biomarkers to improve patient stratification and management(8). The innovation in this project is to extend this technology to include discoveries from WGS and integrate them with quantitative MRI and histopathology data to produce a software product for clinical deployment to inform the management of patients with liver tumours. This approach would provide clinicians with a 'micro to macro' approach to cancer management, identifying genetic variants associated with tumour pathology, liver function, and treatment efficacy, along with their association with pathological and radiological features that can be readily characterised in routine clinical practice. The complementarity of imaging with WGS will be captured by evaluating the current state of the liver from imaging and the future potential of the liver tissue to impact post-operative recovery such as genetic markers for reduced liver regeneration.

For peer review only

Methods and Analysis

Study design:

A prospective observational cohort study.

Study Objectives and Endpoints

Primary Objective

- To determine the utility of whole genome sequencing (WGS) to aid clinical decision making in patients referred for liver resection.

Secondary Objectives

1. To determine the utility of quantitative multiparametric MRI with Liver*MultiScan* to aid clinical decision making in patients referred for liver resection.
2. To compare computationally-derived digital pathology results with human pathologist assessments of the tumour and non-tumour tissue.
3. To compare histopathological assessments of liver fat and fibro- inflammation with quantitative MRI metrics (cT1, PDFF)
4. To evaluate the long term outcomes and recurrence rates and recurrence patterns of patients as it relates to imaging and whole genome sequencing.
5. To evaluate whether whole genome sequencing enables better stratification of patients prior to surgery.

For peer review only - http://bmjopen.bmj.com/site/about/guidelines.xhtml

Primary Endpoint

- Proportion of patients for whom clinically-actionable data is provided by whole genome sequencing at the time of surgery. This will be evaluated retrospectively, with clinically-actionable data defined as data which would result in a clinician choosing a different medical intervention to the current standard of care.

Secondary Endpoints

1. Proportion of patients for whom clinically-actionable data is provided by Liver*MultiScan*.
2. Correlation of computationally-derived digital pathology results with human pathologist assessments of the tumour and non-tumour tissue, along with assessment of intra- and inter-rater variability.
3. Correlation of MR measurements of steatosis and fibroinflammation with digital pathology and human pathology.
4. Performance of WGS and Liver*MultiScan* for predicting post-surgery length of stay in hospital, post-operative liver function, 1-year mortality and recurrence rates.
5. Proportion of patients for whom actionable biomarkers of drug sensitivity are identified with WGS.

<u>Patient population</u>

For peer review only

Patients due to undergo surgery for suspected liver tumour (primary or secondary). A sample size of up to 200 participants will be recruited from Hampshire Hospitals NHS Foundation Trust.

Inclusion criteria

- Male or female 18 years of age and older willing and able to give informed consent to participate in the study
- Patients being considered for liver resection for primary or secondary liver cancer.

Exclusion Criteria

- The participant may not enter the study with any known contraindication to magnetic resonance imaging (including but not limited to pregnancy, a pacemaker or other metallic unfixed implanted device, metallic fragments, extensive tattoos, severe claustrophobia).
- Any other cause, including a significant underlying disease or disorder which, in the opinion of the investigator, may put the participant at risk by participating in the study or limit the participant's ability to participate.

Study Assessments and data collection

Clinic assessment

For peer review only

For peer review only - http://bmjopen.bmj.com/site/about/guidelines.xhtml

Minimal medical history data will be collected including information regarding previous diagnosis of live cancer, cirrhosis and comorbidities including chronic kidney disease and diabetes. Information about alcohol consumption, smoking and coffee intake will be collected. Information about any recent chemotherapy regimens will be collected.

Blood tests

A blood sample will be collected as per routine clinical care, and blood results will be noted regarding biochemistry, renal profile, liver function tests, haematology, clotting, tumour markers including carcinoembryonic antigen, alpha fetoprotein and CA19-9.

MRI

Current routine clinical practice requests patients to visit the hospital 3 days prior to the planned surgery date for a COVID-19 test. At this visit, participants will be invited for an abdominal non-contrast enhanced MRI scan. Images will be processed to generate metrics indicating the liver tissue characteristics (Liver*MultiScan*) and future liver remnant volume (Hepatica).

Impact of imaging on treatment decision making

The clinical utility of quantitative MRI will be assessed through a survey of the consultant surgeons. The surgical plan prior to and after viewing the quantitative imaging reports will be noted, along with an indication as to the reason for any change in surgical plan with an expectation as to the impact on patient outcome:

For peer review only - http://bmjopen.bmj.com/site/about/guidelines.xhtml

- Improved health of residual liver
- Probability of post-operative complications
- Shorter recovery time

Tissue sample collection

During the surgical resection operation, a sample of tumour tissue will be stored in two manners: fresh frozen and formalin fixed/paraffin embedded. This tissue will be stored locally and then transported for tissue processing and WGS by an approved vendor.

Whole Genome Sequencing

Genetic information from tumour tissue and from participant blood sample will be examined. A case review of all participants will be performed with this data to evaluate the clinically actionable information and what steps would have been taken by the clinical care team if this information were available prior to the operation.

Digital Pathology

Tissue explant samples will be analysed by the clinical histopathologist, and relevant information will be noted. Glass slides will be scanned and analysed using digital pathology methods to evaluate objective scores of fibrosis, inflammation, ballooning and steatosis.

Safety outcomes

For peer review only - http://bmjopen.bmj.com/site/about/guidelines.xhtml

No serious adverse events are anticipated as this study has been designed with minimal risks in mind, especially given the reduced healthcare capacity to deal with adverse events.

Incidental findings

Any incidental findings will be reviewed by a radiologist and assessed by the clinical study team. If any structural organ abnormalities are found in the MRI scan or blood tests, a follow-up/note will be added to the participant's medical record as this may have implications for the participant. The participant can seek counselling/support for any potential abnormal findings from their primary healthcare provider should they wish to.

The Chief Investigator and/or Principal Investigator(s) will subsequently be pleased to discuss medical findings directly with the primary health care provider, if requested.

Statistical analysis plan

The data will be evaluated to address the primary endpoint to calculate the proportion of patients in which the results from WGS would have resulted in a change in clinical management. The change in intended management after WGS results will be modelled as a binary variable on the basis of a binomial distribution.

From the literature, we estimate a prevalence of actionable biomarkers derived from genetics of 31%. Using a 5% allowable margin of error and 95% confidence, a sample

For peer review only

For peer review only - http://bmjopen.bmj.com/site/about/guidelines.xhtml

of 200 is required to give a reliable sample to achieve a change in management of 25% of patients.

Descriptive statistics will be reported for all endpoints specified and any interim analyses. Normality of the data will be determined by use of the Shapiro-Wilk test and visual study of histograms. Results will be expressed as mean with standard deviation for continuous normally distributed variables, as median and interquartile range for non- normally distributed data, and as counts and/or percentages for categorical variables. Linearity of continuous data will be assessed by scatter plots. To explore any potential systematic biases, categorial and demographic characteristics expressed as number (%) will be compared using Chi-square. For all tests, a P <0.05 will indicate statistical significance.

Correlations between results from Liver*MultiScan*/Hepatica-derived quantitative MRI metrics and histological features, as well as results from Liver*MultiScan* and genomic features will be determined with Spearman's rank correlation coefficient and diagnostic performance will be assessed by calculation of the receiver operating characteristic and determination of the area under the curve (AUROC) with 95% confidence intervals (CI). For all tests, a P <0.05 will be taken to indicate statistical significance.

To explore impact of genetic variants associated with liver disease, the liver MR derived biomarkers in those with and without known genetic variants associated with liver disease will be compared using a paired t-test (or non-parametric alternative).

For peer review only

For peer review only - http://bmjopen.bmj.com/site/about/guidelines.xhtml

Radiogenomic exploratory analysis will also be conducted to examine correlations between image-derived biomarkers, genetic markers of organ damage or tumour malignancy. Digital pathology and circulation blood derived biomarkers of disease will be explored with Spearman's Rho (rs) with pairwise comparison conducted using two-sided Kolmogorov-Smirnov (KS) tests. List-wise deletion will be employed per analysis, to included data sets with complete cases for all parameters in the model.

Patient and public involvement statement

No formal patient advisory committee was set up and there was no patient or public involvement in the design and planning of the study. A PPIE group has been assembled and will be consulted during dissemination of the findings.

For peer review only

For peer review only - http://bmjopen.bmj.com/site/about/guidelines.xhtml

Footnotes

Ethics and dissemination

This study was reviewed by the NHS Health Research Authority and given favourable opinion by the Brighton and Sussex Research Ethics Committee (REC Reference 20/PR/0222).

Research findings will be discussed with a Patient and Public Involvement and Engagement group, presented at relevant scientific conferences, and published in open access journals.

Peer review

The project plan was also externally peer reviewed by five assessors from InnovateUK.

Funding statement

This work was supported by InnovateUK grant number 50234.

Competing interests statement

JC, MK, AF, VT, EB, SK, DA, CL, JMB and RB work at and are shareholders of Perspectum Ltd.

Patient consent

For peer review only

All potential participants will be allowed as much time as they need to consider their participation in the study. They will have an opportunity to discuss the study with a local investigator prior to giving informed consent. If they decide to proceed, informed consent will be taken during the first study visit. Participant identifiable information will be removed to ensure anonymity.

Data sharing statement

The final trial dataset will be available to study investigators and the Research Ethic Boards at all participating sites.

Additional investigation team members

Jo McClintock[1], Karen Scott[1], Rekha Rana[1], Hannah Stewart[1], Velko Tonev[2], Alessandro Fichera[2], Elena Bielli[2], Luis Núñez[2], Sina Knapp[2], Debbie Atkinson[2], Caitlin Langford[2], Soubera Rymell[2], J Michael Brady[2], Sarah Larkin[3], Naser Ansari-Pour[3], Mohammad Kazaroun[3].

[1] Department of Hepatobiliary Surgery, Basingstoke and North Hampshire Hospital, Basingstoke, Hampshire, United Kingdom.

[1] Perspectum Ltd Gemini One, 5520 John Smith Drive, Oxford, United Kingdom.

[3] University of Oxford, Oxford, United Kingdom.

For peer review only

Authors' contributions

The study concept and design were conceived by FW, JC, MK, SG, RB, MR. FW, MR will conduct recruitment and data collection. Analysis and interpretation will be performed by FW, JC, MK, SG, RB, MR. JC prepared the first draft of the manuscript. All authors provided edits and critiqued the manuscript for intellectual content.

For peer review only

For peer review only - http://bmjopen.bmj.com/site/about/guidelines.xhtml

References

1. Perspectum. Precision Medicine for Liver Tumours With Quantitative Magnetic Resonance Imaging and Whole Genome Sequencing [Internet]. clinicaltrials.gov; 2021 May [cited 2021 Aug 8]. Report No.: NCT04597710. Available from: https://clinicaltrials.gov/ct2/show/NCT04597710

2. Brothwell MRS, West CM, Dunning AM, Burnet NG, Barnett GC. Radiogenomics in the Era of Advanced Radiotherapy. Clin Oncol. 2019 May 1;31(5):319–25.

3. Sorace J, Aberle DR, Elimam D, Lawvere S, Tawfik O, Wallace WD. Integrating pathology and radiology disciplines: an emerging opportunity? BMC Med. 2012 Sep 5;10:100.

4. Tan MP, Ong EM, Amy D, Tot T. Integrating anatomy, radiology, pathology, and surgery: An alternative approach in resecting multifocal and multicentric breast carcinoma. Breast J. 2017 Nov;23(6):663–9.

5. Fujimoto A, Furuta M, Totoki Y, Tsunoda T, Kato M, Shiraishi Y, et al. Whole-genome mutational landscape and characterization of noncoding and structural mutations in liver cancer. Nat Genet. 2016 May;48(5):500–9.

6. Priestley P, Baber J, Lolkema MP, Steeghs N, de Bruijn E, Shale C, et al. Pan-cancer whole-genome analyses of metastatic solid tumours. Nature. 2019 Nov;575(7781):210–6.

7. Banerjee R, Pavlides M, Tunnicliffe EM, Piechnik SK, Sarania N, Philips R, et al. Multiparametric magnetic resonance for the non-invasive diagnosis of liver disease. J Hepatol. 2014 Jan;60(1):69–77.

8. Pavlides M, Banerjee R, Tunnicliffe EM, Kelly C, Collier J, Wang LM, et al. Multiparametric magnetic resonance imaging for the assessment of non-

For peer review only

alcoholic fatty liver disease severity. Liver Int Off J Int Assoc Study Liver. 2017;37(7):1065–73.

9. Hutton C, Gyngell ML, Milanesi M, Bagur A, Brady M. Validation of a standardized MRI method for liver fat and T2* quantification. PLOS ONE. 2018 Sep 20;13(9):e0204175.

10. Mole DJ, Fallowfield JA, Kendall TJ, Welsh F, Semple SI, Bachtiar V, et al. Study protocol: HepaT1ca - an observational clinical cohort study to quantify liver health in surgical candidates for liver malignancies. BMC Cancer. 2018 Sep 12;18(1):890.

11. Mole DJ, Fallowfield JA, Sherif AE, Kendall T, Semple S, Kelly M, et al. Quantitative magnetic resonance imaging predicts individual future liver performance after liver resection for cancer. PloS One. 2020;15(12):e0238568.

12. Sethi P, Thavanesan N, Welsh FK, Connell J, Pickles E, Kelly M, et al. Quantitative multiparametric MRI allows safe surgical planning in patients undergoing liver resection for colorectal liver metastases: report of two patients. BJR Case Rep. 2021 May 1;7(3):20200172.

For peer review only

STROBE Statement—Checklist of items that should be included in reports of ***cohort studies***

	Item No	Recommendation	Page No
**Title and abstract**	1	(*a*) Indicate the study's design with a commonly used term in the title or the abstract	1
		(*b*) Provide in the abstract an informative and balanced summary of what was done and what was found	2
**Introduction**			
Background/rationale	2	Explain the scientific background and rationale for the investigation being reported	4,5
Objectives	3	State specific objectives, including any prespecified hypotheses	6,7
**Methods**			
Study design	4	Present key elements of study design early in the paper	6
Setting	5	Describe the setting, locations, and relevant dates, including periods of recruitment, exposure, follow-up, and data collection	7
Participants	6	(*a*) Give the eligibility criteria, and the sources and methods of selection of participants. Describe methods of follow-up	7
		(*b*) For matched studies, give matching criteria and number of exposed and unexposed	
Variables	7	Clearly define all outcomes, exposures, predictors, potential confounders, and effect modifiers. Give diagnostic criteria, if applicable	6,7
Data sources/ measurement	8*	For each variable of interest, give sources of data and details of methods of assessment (measurement). Describe comparability of assessment methods if there is more than one group	8,9
Bias	9	Describe any efforts to address potential sources of bias	10
Study size	10	Explain how the study size was arrived at	10
Quantitative variables	11	Explain how quantitative variables were handled in the analyses. If applicable, describe which groupings were chosen and why	10
Statistical methods	12	(*a*) Describe all statistical methods, including those used to control for confounding	10,11
		(*b*) Describe any methods used to examine subgroups and interactions	
		(*c*) Explain how missing data were addressed	
		(*d*) If applicable, explain how loss to follow-up was addressed	
		(*e*) Describe any sensitivity analyses	
**Results**			
Participants	13*	(a) Report numbers of individuals at each stage of study—eg numbers potentially eligible, examined for eligibility, confirmed eligible, included in the study, completing follow-up, and analysed	n/a
		(b) Give reasons for non-participation at each stage	
		(c) Consider use of a flow diagram	
Descriptive data	14*	(a) Give characteristics of study participants (eg demographic, clinical, social) and information on exposures and potential confounders	n/a
		(b) Indicate number of participants with missing data for each variable of interest	
		(c) Summarise follow-up time (eg, average and total amount)	
Outcome data	15*	Report numbers of outcome events or summary measures over time	n/a

For peer review only - http://bmjopen.bmj.com/site/about/guidelines.xhtml

Main results	16	(*a*) Give unadjusted estimates and, if applicable, confounder-adjusted estimates and their precision (eg, 95% confidence interval). Make clear which confounders were adjusted for and why they were included<br>(*b*) Report category boundaries when continuous variables were categorized<br>(*c*) If relevant, consider translating estimates of relative risk into absolute risk for a meaningful time period	n/a
Other analyses	17	Report other analyses done—eg analyses of subgroups and interactions, and sensitivity analyses	n/a
**Discussion**			
Key results	18	Summarise key results with reference to study objectives	n/a
Limitations	19	Discuss limitations of the study, taking into account sources of potential bias or imprecision. Discuss both direction and magnitude of any potential bias	n/a
Interpretation	20	Give a cautious overall interpretation of results considering objectives, limitations, multiplicity of analyses, results from similar studies, and other relevant evidence	n/a
Generalisability	21	Discuss the generalisability (external validity) of the study results	n/a
**Other information**			
Funding	22	Give the source of funding and the role of the funders for the present study and, if applicable, for the original study on which the present article is based	12

*Give information separately for exposed and unexposed groups.

Note: An Explanation and Elaboration article discusses each checklist item and gives methodological background and published examples of transparent reporting. The STROBE checklist is best used in conjunction with this article (freely available on the Web sites of PLoS Medicine at http://www.plosmedicine.org/, Annals of Internal Medicine at http://www.annals.org/, and Epidemiology at http://www.epidem.com/). Information on the STROBE Initiative is available at http://www.strobe-statement.org.

For peer review only

For peer review only - http://bmjopen.bmj.com/site/about/guidelines.xhtml

BMJ Open

Precision medicine for liver tumours with quantitative magnetic resonance imaging and whole genome sequencing (Precision1 trial): Study protocol for observational cohort study.

Journal:	*BMJ Open*
Manuscript ID	bmjopen-2021-057163.R1
Article Type:	Protocol
Date Submitted by the Author:	21-Jan-2022
Complete List of Authors:	Welsh, Fenella; Basingstoke and North Hampshire Hospital, Department of Hepatobiliary Surgery<br>Connell, John; Perspectum Diagnostics Ltd<br>Kelly, Matthew; Perspectum Diagnostics Ltd, Innovation<br>Gooding, Sarah; University of Oxford<br>Banerjee, Rajarshi; Perspectum Diagnostics Ltd<br>Rees, Myrddin; Basingstoke and North Hampshire Hospital, Department of Hepatobiliary Surgery
**Primary Subject Heading**:	Oncology
Secondary Subject Heading:	Surgery, Radiology and imaging, Genetics and genomics
Keywords:	Hepatobiliary surgery < SURGERY, Magnetic resonance imaging < RADIOLOGY & IMAGING, GENETICS

SCHOLARONE™
Manuscripts

Precision medicine for liver tumours with quantitative magnetic resonance imaging and whole genome sequencing (Precision1 trial): Study protocol for observational cohort study.

Fenella KS Welsh[1], John J Connell[2], Matthew Kelly[2], Sarah Gooding[3], Rajarshi Banerjee[2], Myrddin Rees[1]

[1] Department of Hepatobiliary Surgery, Basingstoke and North Hampshire Hospital, Basingstoke, Hampshire, United Kingdom.

[2] Perspectum Ltd Gemini One, 5520 John Smith Drive, Oxford, United Kingdom.

[3] University of Oxford, Oxford, United Kingdom.

Corresponding author:

Dr John Connell

Perspectum Ltd

Gemini One, 5520 John Smith Drive

Oxford

For peer review only

OX4 2LL

john.connell@perspectum.com

+44 (0) 1865 655 343

For peer review only

For peer review only - http://bmjopen.bmj.com/site/about/guidelines.xhtml

Abstract

Introduction

Radiogenomic analysis of patients being considered for liver resection is seldom performed in the clinic despite recent evidence indicating that quantitative MRI could improve post-hepatectomy outcomes. Meanwhile, the increasingly accessible results from whole genome sequencing reporting on clinically actionable genetic biomarkers is yet to be fully integrated into the clinical care pathway.

Methods and analysis

A prospective observational cohort study of up to 200 participants is planned, recruiting adults with primary or secondary liver cancer being considered for liver resection at Hampshire Hospitals NHS Foundation Trust. The data will be evaluated to address the primary endpoint to calculate the proportion of participants in which the results from whole genome sequencing would have resulted in a change in clinical management. Participants will be offered an additional non-invasive quantitative MRI scan prior to the operation and the impact of the imaging results on treatment decision making will be evaluated.

Ethics and dissemination

This study was reviewed by the NHS Health Research Authority and given favourable opinion by the Brighton and Sussex Research Ethics Committee (REC Reference

For peer review only

For peer review only - http://bmjopen.bmj.com/site/about/guidelines.xhtml

20/PR/0222). Research findings will be discussed with a Patient and Public Involvement and Engagement group, presented at relevant scientific conferences and published in open access journals.

Trial Registration

Clinicaltrials.gov identifier: NCT04597710

Strengths and limitations of this study

- Precision1 will be the first study examining the clinical utility of radiogenomic information during the surgical treatment planning stage for patients with primary and secondary liver cancer
- Our prospective study is designed to allow us to understand the extent to which quantitative MRI of the liver impacts surgical treatment planning
- The additional value of whole genome sequencing will be evaluated to help us to identify the presence of any clinically actionable genetic biomarkers which may support precision medicine provision to patients.
- All patients being considered for liver resection may be eligible for the study, regardless of tumour type or clinical background.
- The primary limitation of this study is the omission of a tightly matched negative control group where the impact of quantitative imaging on improving patient outcomes could be rigorously examined.

For peer review only

Introduction

The incidence of treatable liver tumours is on the rise globally, driven by obesity, viral hepatitis and metastases from colorectal cancers. Survival rates can be improved with optimised allocation of treatment options including surgical resection, radiofrequency ablation, embolisation, chemotherapy and targeted molecular therapies (including immunotherapy). The key motivation of this study is to help patients access the most suitable treatment combinations, by integrating radiogenomic(1) and clinical data.

A similar integrated approach, integrating radiology and pathology, has been shown to improve outcomes in breast cancer care(2). Detailed pathologic analysis of the surgical specimen from breast carcinoma biopsy provides valuable feedback to the radiologist, establishes the completeness of surgical intervention, and generates predictive information for therapeutic decisions(3).

Whole genome sequencing (WGS) has revealed cancer driver mutations and the complex molecular profile of liver cancer(4). In many metastatic solid tumours, WGS has been used to identify a significant patient population (31%) who present with a biomarker that predicts sensitivity to a drug and lack any known resistance biomarkers for the same drug(5). Identifying which patients possess genomic variants that predict drug response or resistance will allow clinicians to make the optimal treatment decisions. The next technological challenge is integrating WGS into scalable clinical practice.

For peer review only

In addition to the molecular profile of the patient's liver cancer, a non-invasive assessment of the overall health of the liver using quantitative MRI imaging will be carried out in this study. The MRI metrics from Liver*MultiScan*® can quantify changes to liver tissue characteristics, including fibro-inflammation and fat, with the accuracy and reproducibility of the results demonstrated in a number of studies(6–8). A recent InnovateUK grant supported Perspectum, Hampshire Hospitals NHS Foundation Trust (HHFT) and University of Edinburgh to assess the use of quantitative MRI in decision support for liver resection. This prospective 143 patient study showed quantitative MRI using Hepatica™ (which incorporates Liver*MultiScan*®) can predict length of patient stay(9–11). Patients with liver cancer plus often undiagnosed chronic liver disease performed worse than those with healthy native livers; they also recovered more slowly, costing an extra 1.5 days of hospital stay per patient, a significant clinical impact.

The gold standard of liver assessment relies on imaging and biopsies. Advances in quantitative algorithms provide richer data about the cellular changes associated with liver disease, and greater insight into disease monitoring and treatment efficacy from MRI and digital pathology. In this project, by additionally analysing the genome of both tumour and germline, we aim to understand which variants are driving tumour growth and potentially amenable to targeted therapies and impacting treatment response, along with variants associated with response to surgery and liver regeneration.

For peer review only - http://bmjopen.bmj.com/site/about/guidelines.xhtml

In previous work we have demonstrated the ability to combine MRI imaging, liver histopathology, and circulating (enzyme) biomarkers to improve patient stratification and management(7). The innovation in this project is to extend this technology to include discoveries from WGS and integrate them with quantitative MRI and histopathology data to produce a software product for clinical deployment to inform the management of patients with liver tumours. This approach would provide clinicians with a 'micro to macro' approach to cancer management, identifying genetic variants associated with tumour pathology, liver function, and treatment efficacy, along with their association with pathological and radiological features that can be readily characterised in routine clinical practice. The complementarity of imaging with WGS will be captured by evaluating the current state of the liver from imaging and the future potential of the liver tissue to impact post-operative recovery such as genetic markers for reduced liver regeneration.

For peer review only

For peer review only - http://bmjopen.bmj.com/site/about/guidelines.xhtml

Methods and Analysis

Study design:

A prospective observational cohort study.

Study Objectives and Endpoints

Primary Objective

- To determine the utility of whole genome sequencing (WGS) to aid clinical decision making in patients referred for liver resection.

Secondary Objectives

1. To determine the utility of quantitative multiparametric MRI with Liver*MultiScan* to aid clinical decision making in patients referred for liver resection.
2. To compare computationally-derived digital pathology results with human pathologist assessments of the tumour and non-tumour tissue.
3. To compare histopathological assessments of liver fat and fibro- inflammation with quantitative MRI metrics (cT1, PDFF)
4. To evaluate the long term outcomes and recurrence rates and recurrence patterns of patients as it relates to imaging and whole genome sequencing.
5. To evaluate whether whole genome sequencing enables better stratification of patients prior to surgery.

For peer review only - http://bmjopen.bmj.com/site/about/guidelines.xhtml

Primary Endpoint

- Proportion of patients for whom clinically-actionable data is provided by whole genome sequencing at the time of surgery. This will be evaluated retrospectively, with clinically-actionable data defined as data which would result in a clinician choosing a different medical intervention to the current standard of care.

Secondary Endpoints

1. Proportion of patients for whom clinically-actionable data is provided by Liver*MultiScan*.
2. Correlation of computationally-derived digital pathology results with human pathologist assessments of the tumour and non-tumour tissue, along with assessment of intra- and inter-rater variability.
3. Correlation of MR measurements of steatosis and fibroinflammation with digital pathology and human pathology.
4. Performance of WGS and Liver*MultiScan* for predicting post-surgery length of stay in hospital, post-operative liver function, 1-year mortality and recurrence rates.
5. Proportion of patients for whom actionable biomarkers of drug sensitivity are identified with WGS.

<u>Patient population</u>

For peer review only

Patients due to undergo surgery for suspected liver tumour (primary or secondary). A sample size of up to 200 participants will be recruited from Hampshire Hospitals NHS Foundation Trust.

Inclusion criteria

- Male or female 18 years of age and older willing and able to give informed consent to participate in the study
- Patients being considered for liver resection for primary or secondary liver cancer.

Exclusion Criteria

- The participant may not enter the study with any known contraindication to magnetic resonance imaging (including but not limited to pregnancy, a pacemaker or other metallic unfixed implanted device, metallic fragments, extensive tattoos, severe claustrophobia).
- Any other cause, including a significant underlying disease or disorder which, in the opinion of the investigator, may put the participant at risk by participating in the study or limit the participant's ability to participate.

Study Assessments and data collection

Study visits are summarised in Figure 1.

Clinic assessment

For peer review only

For peer review only - http://bmjopen.bmj.com/site/about/guidelines.xhtml

Minimal medical history data will be collected including information regarding previous diagnosis of live cancer, cirrhosis and comorbidities including chronic kidney disease and diabetes. Information about alcohol consumption, smoking and coffee intake will be collected. Information about any recent chemotherapy regimens will be collected.

<u>Blood tests</u>

A blood sample will be collected as per routine clinical care, and blood results will be noted regarding biochemistry, renal profile, liver function tests, haematology, clotting, tumour markers including carcinoembryonic antigen, alpha fetoprotein and CA19-9.

<u>MRI</u>

Current routine clinical practice requests patients to visit the hospital 3 days prior to the planned surgery date for a COVID-19 test. At this visit, participants will be invited for an abdominal non-contrast enhanced MRI scan. Images will be processed to generate metrics indicating the liver tissue characteristics (Liver*MultiScan*) and future liver remnant volume (Hepatica) as previously described(12).

<u>Impact of imaging on treatment decision making</u>

The clinical utility of quantitative MRI will be assessed through a survey of the consultant surgeons. The surgical plan prior to and after viewing the quantitative imaging reports will be noted, along with an indication as to the reason for any change in surgical plan with an expectation as to the impact on patient outcome:

For peer review only

- Improved health of residual liver
- Probability of post-operative complications
- Shorter recovery time

Tissue sample collection

During the surgical resection operation, a sample of tumour tissue from the explant will be stored in two manners: fresh frozen and formalin fixed/paraffin embedded. This tissue and a blood sample will be stored locally and then transported for tissue processing and WGS by an approved vendor.

Whole Genome Sequencing

Genetic information from tumour tissue and from participant blood sample will be examined. A case review of all participants will be performed with this data to evaluate the clinically actionable information and what steps would have been taken by the clinical care team if this information were available prior to the operation.

Digital Pathology

Tissue explant samples will be analysed by the clinical histopathologist, and relevant information will be noted. Glass slides will be scanned and analysed using digital pathology methods to evaluate objective scores of fibrosis, inflammation, ballooning and steatosis.

Safety outcomes

For peer review only

No serious adverse events are anticipated as this study has been designed with minimal risks in mind, especially given the reduced healthcare capacity to deal with adverse events.

Incidental findings

Any incidental findings will be reviewed by a radiologist and assessed by the clinical study team. If any structural organ abnormalities are found in the MRI scan or blood tests, a follow-up/note will be added to the participant's medical record as this may have implications for the participant. The participant can seek counselling/support for any potential abnormal findings from their primary healthcare provider should they wish to.

The Chief Investigator and/or Principal Investigator(s) will subsequently be pleased to discuss medical findings directly with the primary health care provider, if requested.

Statistical analysis plan

The data will be evaluated to address the primary endpoint to calculate the proportion of patients in which the results from WGS would have resulted in a change in clinical management. The change in intended management after WGS results will be modelled as a binary variable on the basis of a binomial distribution.

From the literature, we estimate a prevalence of actionable biomarkers derived from genetics of 31%. Using a 5% allowable margin of error and 95% confidence, a sample

For peer review only

of 200 is required to give a reliable sample to achieve a change in management of 25% of patients.

Descriptive statistics will be reported for all endpoints specified and any interim analyses. Normality of the data will be determined by use of the Shapiro-Wilk test and visual study of histograms. Results will be expressed as mean with standard deviation for continuous normally distributed variables, as median and interquartile range for non- normally distributed data, and as counts and/or percentages for categorical variables. Linearity of continuous data will be assessed by scatter plots. To explore any potential systematic biases, categorial and demographic characteristics expressed as number (%) will be compared using Chi-square. For all tests, a P <0.05 will indicate statistical significance.

Correlations between results from Liver*MultiScan*/Hepatica-derived quantitative MRI metrics and histological features, as well as results from Liver*MultiScan* and genomic features will be determined with Spearman's rank correlation coefficient and diagnostic performance will be assessed by calculation of the receiver operating characteristic and determination of the area under the curve (AUROC) with 95% confidence intervals (CI). For all tests, a P <0.05 will be taken to indicate statistical significance.

To explore impact of genetic variants associated with liver disease, the liver MR derived biomarkers in those with and without known genetic variants associated with liver disease will be compared using a paired t-test (or non-parametric alternative).

For peer review only

For peer review only - http://bmjopen.bmj.com/site/about/guidelines.xhtml

Radiogenomic exploratory analysis will also be conducted to examine correlations between image-derived biomarkers, genetic markers of organ damage or tumour malignancy. Digital pathology and circulation blood derived biomarkers of disease will be explored with Spearman's Rho (rs) with pairwise comparison conducted using two-sided Kolmogorov-Smirnov (KS) tests. List-wise deletion will be employed per analysis, to included data sets with complete cases for all parameters in the model.

Patient and public involvement statement

No formal patient advisory committee was set up and there was no patient or public involvement in the design and planning of the study. A PPIE group has been assembled and will be consulted during dissemination of the findings.

For peer review only

For peer review only - http://bmjopen.bmj.com/site/about/guidelines.xhtml

Footnotes

Ethics and dissemination

This study was reviewed by the NHS Health Research Authority and given favourable opinion by the Brighton and Sussex Research Ethics Committee (REC Reference 20/PR/0222).

Research findings will be discussed with a Patient and Public Involvement and Engagement group, presented at relevant scientific conferences, and published in open access journals.

Peer review

The project plan was also externally peer reviewed by five assessors from InnovateUK.

Funding statement

This work was supported by InnovateUK grant number 50234.

Competing interests statement

JC, MK, AF, VT, EB, SK, DA, CL, JMB and RB work at and are shareholders of Perspectum Ltd.

Patient consent

For peer review only

All potential participants will be allowed as much time as they need to consider their participation in the study. They will have an opportunity to discuss the study with a local investigator prior to giving informed consent. If they decide to proceed, informed consent will be taken during the first study visit. Participant identifiable information will be removed to ensure anonymity.

Data sharing statement

The final trial dataset will be available to study investigators and the Research Ethic Boards at all participating sites. A full data management plan is detailed within the trial protocol. This indicates definitions of source data, rules governing access to data, pseudonymisation process, document storage, data security and privacy compliance, and data transfer methods.

Additional investigation team members

Jo McClintock[1], Karen Scott[1], Rekha Rana[1], Hannah Stewart[1], Velko Tonev[2], Alessandro Fichera[2], Elena Bielli[2], Luis Núñez[2], Sina Knapp[2], Debbie Atkinson[2], Caitlin Langford[2], Soubera Rymell[2], J Michael Brady[2], Sarah Larkin[3], Naser Ansari-Pour[3], Mohammad Kazaroun[3].

[1] Department of Hepatobiliary Surgery, Basingstoke and North Hampshire Hospital, Basingstoke, Hampshire, United Kingdom.

[1] Perspectum Ltd Gemini One, 5520 John Smith Drive, Oxford, United Kingdom.

For peer review only

[3] University of Oxford, Oxford, United Kingdom.

Authors’ contributions

The study concept and design were conceived by FW, JC, MK, SG, RB, MR. FW, MR will conduct recruitment and data collection. Analysis and interpretation will be performed by FW, JC, MK, SG, RB, MR. JC prepared the first draft of the manuscript. All authors provided edits and critiqued the manuscript for intellectual content.

Figure 1

Study flow chart. Participants with informed consent will undergo pre-treatment measurements at Study Visit 1 including height & weight, blood pressure, an online questionnaire, a non-contrast MRI scan and standard liver blood tests. Standard-of-care treatment will be followed and a blood sample and a sample tumour tissue explant will be collected. 12 months following recruitment, a post-treatment telephone interview will occur as Study Visit 2.

For peer review only

References

1. Brothwell MRS, West CM, Dunning AM, Burnet NG, Barnett GC. Radiogenomics in the Era of Advanced Radiotherapy. Clin Oncol. 2019 May 1;31(5):319–25.

2. Sorace J, Aberle DR, Elimam D, Lawvere S, Tawfik O, Wallace WD. Integrating pathology and radiology disciplines: an emerging opportunity? BMC Med. 2012 Sep 5;10:100.

3. Tan MP, Ong EM, Amy D, Tot T. Integrating anatomy, radiology, pathology, and surgery: An alternative approach in resecting multifocal and multicentric breast carcinoma. Breast J. 2017 Nov;23(6):663–9.

4. Fujimoto A, Furuta M, Totoki Y, Tsunoda T, Kato M, Shiraishi Y, et al. Whole-genome mutational landscape and characterization of noncoding and structural mutations in liver cancer. Nat Genet. 2016 May;48(5):500–9.

5. Priestley P, Baber J, Lolkema MP, Steeghs N, de Bruijn E, Shale C, et al. Pan-cancer whole-genome analyses of metastatic solid tumours. Nature. 2019 Nov;575(7781):210–6.

6. Banerjee R, Pavlides M, Tunnicliffe EM, Piechnik SK, Sarania N, Philips R, et al. Multiparametric magnetic resonance for the non-invasive diagnosis of liver disease. J Hepatol. 2014 Jan;60(1):69–77.

7. Pavlides M, Banerjee R, Tunnicliffe EM, Kelly C, Collier J, Wang LM, et al. Multiparametric magnetic resonance imaging for the assessment of non-alcoholic fatty liver disease severity. Liver Int Off J Int Assoc Study Liver. 2017;37(7):1065–73.

8. Hutton C, Gyngell ML, Milanesi M, Bagur A, Brady M. Validation of a standardized MRI method for liver fat and T2* quantification. PLOS ONE. 2018 Sep 20;13(9):e0204175.

For peer review only

9. Mole DJ, Fallowfield JA, Kendall TJ, Welsh F, Semple SI, Bachtiar V, et al. Study protocol: HepaT1ca - an observational clinical cohort study to quantify liver health in surgical candidates for liver malignancies. BMC Cancer. 2018 Sep 12;18(1):890.

10. Mole DJ, Fallowfield JA, Sherif AE, Kendall T, Semple S, Kelly M, et al. Quantitative magnetic resonance imaging predicts individual future liver performance after liver resection for cancer. PloS One. 2020;15(12):e0238568.

11. Sethi P, Thavanesan N, Welsh FK, Connell J, Pickles E, Kelly M, et al. Quantitative multiparametric MRI allows safe surgical planning in patients undergoing liver resection for colorectal liver metastases: report of two patients. BJR Case Rep. 2021 May 1;7(3):20200172.

12. Mojtahed A, Núñez L, Connell J, Fichera A, Nicholls R, Barone A, et al. Repeatability and reproducibility of deep-learning-based liver volume and Couinaud segment volume measurement tool. Abdom Radiol [Internet]. 2021 Oct 4 [cited 2021 Oct 27]; Available from: https://doi.org/10.1007/s00261-021-03262-x

For peer review only

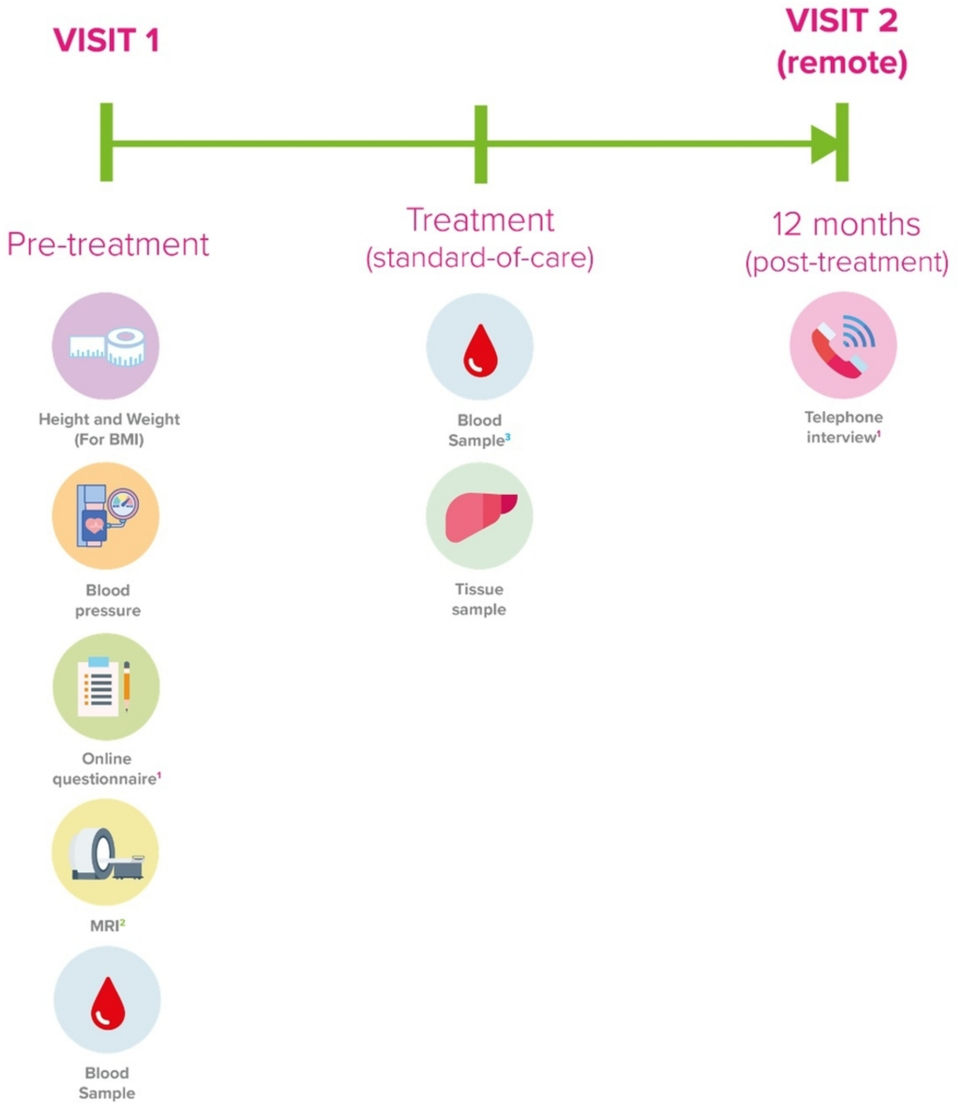

Study flow chart. Participants with informed consent will undergo pre-treatment measurements at Study Visit 1 including height & weight, blood pressure, an online questionnaire, a non-contrast MRI scan and standard liver blood tests. Standard-of-care treatment will be followed and a blood sample and a sample tumour tissue explant will be collected. 12 months following recruitment, a post-treatment telephone interview will occur as Study Visit 2.

90x101mm (300 x 300 DPI)

STROBE Statement—Checklist of items that should be included in reports of ***cohort studies***

	Item No	Recommendation	Page No
**Title and abstract**	1	(*a*) Indicate the study's design with a commonly used term in the title or the abstract	1
		(*b*) Provide in the abstract an informative and balanced summary of what was done and what was found	2
**Introduction**			
Background/rationale	2	Explain the scientific background and rationale for the investigation being reported	4,5
Objectives	3	State specific objectives, including any prespecified hypotheses	6,7
**Methods**			
Study design	4	Present key elements of study design early in the paper	6
Setting	5	Describe the setting, locations, and relevant dates, including periods of recruitment, exposure, follow-up, and data collection	7
Participants	6	(*a*) Give the eligibility criteria, and the sources and methods of selection of participants. Describe methods of follow-up<br>(*b*) For matched studies, give matching criteria and number of exposed and unexposed	7
Variables	7	Clearly define all outcomes, exposures, predictors, potential confounders, and effect modifiers. Give diagnostic criteria, if applicable	6,7
Data sources/ measurement	8*	For each variable of interest, give sources of data and details of methods of assessment (measurement). Describe comparability of assessment methods if there is more than one group	8,9
Bias	9	Describe any efforts to address potential sources of bias	10
Study size	10	Explain how the study size was arrived at	10
Quantitative variables	11	Explain how quantitative variables were handled in the analyses. If applicable, describe which groupings were chosen and why	10
Statistical methods	12	(*a*) Describe all statistical methods, including those used to control for confounding<br>(*b*) Describe any methods used to examine subgroups and interactions<br>(*c*) Explain how missing data were addressed<br>(*d*) If applicable, explain how loss to follow-up was addressed<br>(*e*) Describe any sensitivity analyses	10,11
**Results**			
Participants	13*	(a) Report numbers of individuals at each stage of study—eg numbers potentially eligible, examined for eligibility, confirmed eligible, included in the study, completing follow-up, and analysed<br>(b) Give reasons for non-participation at each stage<br>(c) Consider use of a flow diagram	n/a
Descriptive data	14*	(a) Give characteristics of study participants (eg demographic, clinical, social) and information on exposures and potential confounders<br>(b) Indicate number of participants with missing data for each variable of interest<br>(c) Summarise follow-up time (eg, average and total amount)	n/a
Outcome data	15*	Report numbers of outcome events or summary measures over time	n/a

For peer review only

Main results	16	(*a*) Give unadjusted estimates and, if applicable, confounder-adjusted estimates and their precision (eg, 95% confidence interval). Make clear which confounders were adjusted for and why they were included<br>(*b*) Report category boundaries when continuous variables were categorized<br>(*c*) If relevant, consider translating estimates of relative risk into absolute risk for a meaningful time period	n/a
Other analyses	17	Report other analyses done—eg analyses of subgroups and interactions, and sensitivity analyses	n/a
**Discussion**			
Key results	18	Summarise key results with reference to study objectives	n/a
Limitations	19	Discuss limitations of the study, taking into account sources of potential bias or imprecision. Discuss both direction and magnitude of any potential bias	n/a
Interpretation	20	Give a cautious overall interpretation of results considering objectives, limitations, multiplicity of analyses, results from similar studies, and other relevant evidence	n/a
Generalisability	21	Discuss the generalisability (external validity) of the study results	n/a
**Other information**			
Funding	22	Give the source of funding and the role of the funders for the present study and, if applicable, for the original study on which the present article is based	12

*Give information separately for exposed and unexposed groups.

Note: An Explanation and Elaboration article discusses each checklist item and gives methodological background and published examples of transparent reporting. The STROBE checklist is best used in conjunction with this article (freely available on the Web sites of PLoS Medicine at http://www.plosmedicine.org/, Annals of Internal Medicine at http://www.annals.org/, and Epidemiology at http://www.epidem.com/). Information on the STROBE Initiative is available at http://www.strobe-statement.org.

For peer review only

For peer review only - http://bmjopen.bmj.com/site/about/guidelines.xhtml
